# Gigantic GIST: A Case of the Largest Gastrointestinal Stromal Tumor Found to Date

**DOI:** 10.1155/2018/6170861

**Published:** 2018-09-30

**Authors:** Abdalla Mohamed, Youssef Botros, Paul Hanna, Sang Lee, Walid Baddoura, Jamshed Zuberi, Tanuja Damani

**Affiliations:** ^1^Department of Medicine, St. Joseph's University Medical Center, Paterson, NJ, USA; ^2^Division of Gastroenterology and Hepatology, St. Joseph's University Medical Center, Paterson, NJ, USA; ^3^Department of Surgery, St. Joseph's University Medical Center, Paterson, NJ, USA

## Abstract

Gastrointestinal stromal tumors are uncommon when compared to all gastrointestinal neoplasms but are the most common mesenchymal tumors of the gastrointestinal tract. The largest gastrointestinal stromal tumor ever recorded in literature weighed approximately 6.1 kg and measured 39 cm × 27 cm × 14 cm. About two-thirds of GISTs are malignant. The tumor size, mitotic rate, cellularity, and nuclear pleomorphism are the most important parameters when considering prognosis and recurrence. The definitive treatment for these tumors is resection. In the year 2000, the first patient was treated with the tyrosine kinase inhibitor imatinib and since then, gastrointestinal stromal tumors with high-risk features have been treated successfully with tyrosine kinase inhibitors. We present the largest gastrointestinal stromal tumor recorded in medical literature measuring 42.0 cm × 31.0 cm × 23.0 cm in maximum dimensions and weighing in at approximately 18.5 kg in a 65-year-old African-American male who presented with increased abdominal distention. The mass was successfully excised, and the patient was treated with imatinib without local or distant recurrence 1.5 years postoperatively.

## 1. Introduction

Although GISTs are relatively uncommon (<1% of all gastrointestinal neoplasms) compared with adenocarcinoma, they are the most common mesenchymal tumors of the gastrointestinal tract [1, 2]. According to SEER (Surveillance, Epidemiology, and End Results) data from 1992 to 2000, the estimated incidence in the USA is 6.8 per million. The incidence of occult micro-GISTs smaller than 1 cm is actually much higher [3].

The majority of cases present as symptomatic disease. Small GISTs are usually asymptomatic and are incidentally found during radiologic evaluation, endoscopy, or at the time of surgery for another cause. Although a GIST is not a mucosa-based tumor, like adenocarcinoma, but instead grows from the muscle layer of the bowel wall, it still may be accompanied by bleeding in up to 25% of patients due to erosion of the mucosa resulting in melena, hematemesis, or symptomatic anemia. Other signs and symptoms include abdominal fullness, early satiety, a palpable mass, or abdominal pain [4]. To our knowledge, the largest reported GIST in the literature measured 39 cm × 27 cm × 14 cm and weighed 6.1 kg [5].

Tumor size and the mitotic count are the most commonly used prognostic factors to estimate the outcome and risk of recurrence of the tumor. Tumors arising from the stomach have a more favorable prognosis compared to other sites [6]. Treatment strategy for GIST will depend on the tumor site, size, and presence or absence of metastasis. Small GIST (less than 2 cm) can be conservatively observed [7]. Surgical resection is the mainstay for nonmetastatic tumors, most commonly in the form of a wedge resection. Another modality for treating small tumors is endoscopic resection [8].

We present a rare case of a gastric GIST larger in size than any previously documented, rapidly growing and invading into the surrounding structures that was successfully treated with surgical resection with total gastrectomy as well as en bloc resection of the pancreas, spleen, and colon with ostomy creation.

GISTs are encountered frequently in the surgical clinical setting with varying sizes, invasion, and treatment modalities. We present this case for a review of GISTs and review of the surgical decision-making and to highlight the patient's success despite massive resection of major abdominal organs.

## 2. Case Presentation

A 65-year-old African-American male with no significant past medical history presented to facility with complaints of worsening abdominal distention for approximately one year, associated with dyspnea, early satiety, and weight loss of about 23 pounds. He denied chest pain, melena, or hematochezia. He had never had an endoscopy or colonoscopy performed. The patient did have a remote history of smoking of about a half pack of cigarettes per day for 10 years; however, he had quit 15 years prior. Family history was significant for a brother with lung cancer, otherwise noncontributory.

On physical examination, he was noted to have bitemporal wasting and marked abdominal distension secondary to a large firm mass with ill-defined margins.

**Figure 1:**
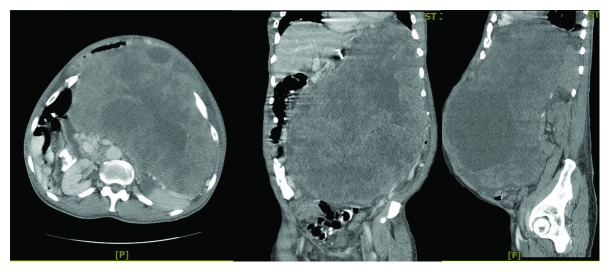
Coronal, transverse, and sagittal views of CT of the abdomen and pelvis showing heterogeneous, partially necrotic mass.

A contrast-enhanced CT scan of the abdomen and pelvis showed a large, heterogeneous, partially necrotic mass measuring 38 cm × 25 cm, arising from the left upper quadrant with no evidence of metastases seen. There was displacement of the stomach and duodenum to the right and the left kidney was displaced inferiorly. The mass was suggestive of a sarcoma or possibly a GIST tumor with malignant degeneration (Figure 1). Chest radiograph was done which confirmed no pulmonary pathology or metastatic disease.

Preoperative laboratory analysis showed a white blood cell count of 5.9 K/mm^3^ with 80% neutrophils. Hemoglobin of 10 G/dl and hematocrit 31.2% with a platelet count of 328 K/mm^3^. Basic metabolic panel and liver function tests and PT/INR were unremarkable.

The patient was taken for an exploratory laparotomy, which revealed a large mass arising from the posterior gastric wall with a giant omental vessel as well as dilated gastroepiploic vessels. The mass was invading the spleen, distal pancreas, and the mesentery of the transverse colon. Over the next 7 hours, the mass was completely resected with a total gastrectomy, esophagojejunostomy and feeding jejunostomy, distal pancreatectomy, and splenectomy. Resection required division of the mesentery of the transverse colon, which resulted in ischemia of the transverse colon warranting resection with end colostomy creation. The tumor, spleen, distal pancreas, and transverse colon were all sent for frozen section. As expected of GISTs, pathology reports showed that the 6 harvested lymph nodes as well as the surgical margins were all negative for malignancy. During the 7-hour procedure, the patient lost nearly 3 l of blood and received 6 l of IV fluids, 12 units of packed red blood cells, 4 units of fresh-frozen plasma, 1 unit of single donor platelets, and 4 units of albumin with intermittent pushes of vasopressive medications. The patient was taken to the surgical intensive care unit postoperatively, was weaned off vasopressive medications, extubated, and started on tube feeds, and eventually fed orally. The remainder of his hospital course was uneventful, and the patient was discharged to home on postoperative day 14. The patient was started on imatinib at this time and has been maintained on that with no recurrence approximately 1.5 years postoperatively. The patient returned 8 months later for a planned colostomy take-down, which was successful and uneventful.

**Figure 2:**
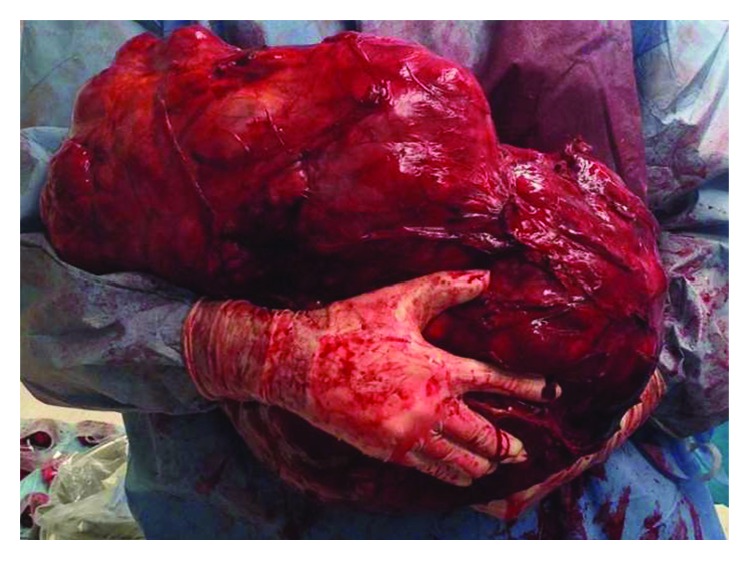
Gross image of the resected mass measuring 42 × 31 × 23 cm and weighing 18.5 kg.

Macroscopically, the mass was irregularly shaped, attached to the posterior-inferior aspect of the stomach, weighed 18,500 grams, and measured 42.0 × 31.0 × 23.0 cm in maximum dimensions (Figure 2).

Histopathology showed gastric stromal tumor cells staged as high grade with >5 mitoses per high power field (hpf).

**Figure 3:**
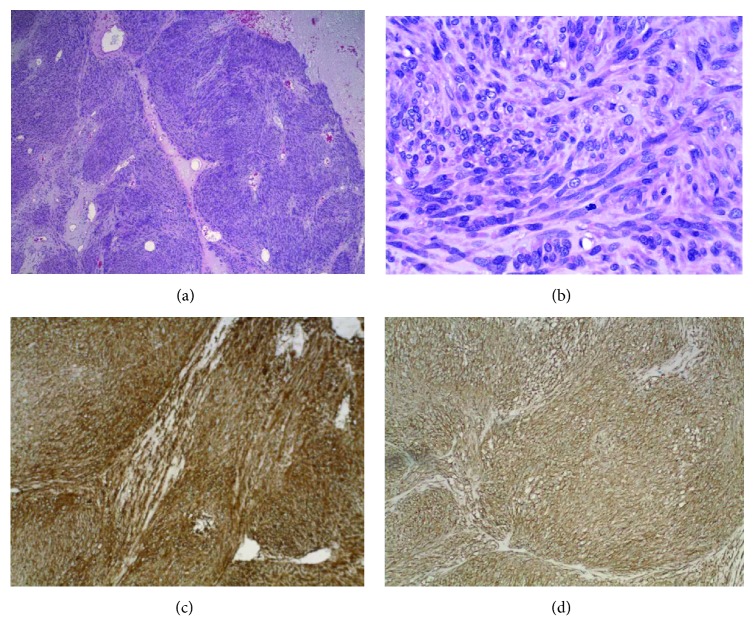
(a) Low-power H&E stain. (b) High-power H&E stain showing gastric stromal tumor cells with mitosis. (c) DOG-1 stain: strongly positive. (d) CD117 stain: strongly positive.

Immunohistochemistry confirmed GIST with strong positive staining for CD117, DOG1, and CD34 (Figure 3). Postoperative recovery was uneventful.

## 3. Discussion

Our patient presented with progressively worsening abdominal distension for 9 months, weight loss, and was found to have a giant GIST causing early satiety, anemia, and locally invading into adjacent organs such as the spleen, distal pancreas, and the mesentery of the transverse colon. Despite the enormous size and weight of the tumor, our patient denied any severe abdominal pain or other associated symptoms other than abdominal distention with decreased diet tolerance.

We report the largest GIST tumor reported to date. The size of our tumor measured 42.0 cm × 31.0 cm × 23.0 cm in dimensions. The largest tumor reported prior to our tumor measured 39 cm × 27 cm × 14 cm. The weight of our tumor was 18.5 kg, which is about 3 times heavier than the heaviest tumor previously reported, 6.1 kg.

The treatment strategy for GIST depends on the tumor site, size, and presence or absence of metastasis. In this case, surgery was unavoidable due to the complex nature and size of the tumor as well as the associated early gastrointestinal obstruction identified by patient's decreased diet tolerance and findings on the imaging. For this reason, the decision was made to proceed with surgical intervention as opposed to neoadjuvant therapy with imatinib. Achieving an adequate en bloc resection was difficult due to the multivisceral involvement. The patient underwent a total gastrectomy, esophagojejunostomy and feeding jejunostomy, distal pancreatectomy, and splenectomy. The added difficulty during the case was after resection of the transverse mesocolon, finding evidence of a nonviable segment of bowel from the transverse colon to mid descending colon which was resected and a temporary ostomy was created. It was not advisable to create a primary anastomosis at this time given the patient's hypotension, significant blood loss, and transfusion requirement. There is no consensus on the use of neoadjuvant therapy for the treatment of GISTs. There have been approximately seven trials in which neoadjuvant chemotherapy was studied and utilized [9]. RTOG (Radiation Therapy Oncology Group) 0132 was the first trial of preoperative imatinib in GIST [9, 10]. Thirty-one primary GIST patients were analyzed as the neoadjuvant group. The median tumor size was 8.7 cm. Imatinib was administered at 600 mg/day for 8 to 12 weeks before surgery, and imatinib administration also continued for 2 years after surgery. In the primary GIST group, the progression-free survival, which was the primary end point of this trial, was calculated as 83.9% for 2 years and 56.7% for 5 years [9, 10]. This was the first trial to show some use of neoadjuvant imatinib, but unfortunately, the same trial failed to show any superiority of adding neoadjuvant chemotherapy compared to adjuvant alone [9, 10]. Kurokawa et al. had similar results in a recent study published in 2017 that showed that neoadjuvant therapy along with surgery may be beneficial long term [10, 11]. As mentioned previously, the decision was made to forego neoadjuvant imatinib therapy due to the development of gastrointestinal obstruction.

As investigated preoperatively, there was also no evidence of hematogenous spread to distal organs on the imaging. As evidenced in previous studies, markers of hepatic invasion by GIST are tumor size of >5 cm and the presence of nodal metastasis or mitotic count greater than 5/hpf [12]. The tumor was confirmed to be a GIST, and approximately 0.5 cm from the closest margin and less than one hpf from the serosa.

Because GISTs often demonstrate an exophytic pattern of growth toward the peritoneal cavity [13], the patient probably remained asymptomatic until the tumor reached a quite large size. Exophytic GISTs can invade structures such as the pancreas or colon and may result in bowel obstruction or adjacent organ dysfunction [13]. Imatinib, a tyrosine kinase inhibitor, could have been used as a neoadjuvant therapy if the tumor pathology was known earlier [10]. Although neoadjuvant therapy may have decreased the magnitude of the surgery, the biopsy would also increase the risk of tumor rupture which is associated with inevitable peritoneal recurrence [14].

Postoperatively, the patient has been monitored for recurrence and basic hematologic parameters because the massive size of the tumor is known to be associated with a higher recurrence rate [13]. Nearly half of patients with GISTs present with metastatic disease, most commonly to the liver and peritoneum [15]. Fortunately, however, our patient's histopathology showed T4N0M0 and pathology from colostomy take-down remained to be negative.

The most important immunohistological features of GIST are as follows: (i) the tumor cells are whorls of spindle-shaped cells with eosinophilic cytoplasm and elongated nuclei; (ii) mitotic figures indicate high risk of progression; and (iii) immunoreactivity to c-KIT, CD117, or DOG1 confirms the diagnosis [16]. Heinrich et al. reported that five percent of GISTs do not demonstrate c-KIT immunoreactivity, and these tumors usually harbor mutations in platelet-derived growth factor receptor [17]. Our immunohistochemistry included DOG1, CD34, CD117, actin, and S100, and the tumor was strongly positive for DOG-1, CD34, and CD117.

In 1998, Hirota et al. described the underlying gain function mutation leading to the development of GIST tumors, the c-kit proto-oncogene (CD117) coding the KIT receptor—a tyrosine kinase transmembrane receptor that controls crucial cell functions in tumor genesis, including proliferation, adhesion, apoptosis, and differentiation. The KIT gene mutation leads to uncontrolled cell proliferation due to stimulation of downstream signaling pathways [18].

Different markers are used to identify KIT-negative GISTs including: calcium-dependent and receptor-activated chloride channel protein, known as DOG1 and protein kinase C theta [19, 20]. Carbonic anhydrase II is another sensitive biomarker for GIST [21]. The importance of distinguishing GIST from other mesenchymal tumors arises from their notorious resistance to conventional chemotherapy and radiation and their good response to the targeted treatment, e.g., imatinib [22].

Adjuvant therapy along with surgery is the preferred method of treatment when it comes to GIST. Imatinib for adjuvant treatment was approved based on the ACOSOG Z9001 study in 2008 in the USA and in 2009 in Europe. In this study, eligible patients had complete resection of a primary GIST of at least 3 cm in size. The median follow-up was 19.7 months. Adjuvant treatment for one year led to a RFS of 98% [23, 24].

Most GIST patients will achieve the clinical benefits with imatinib, but an estimated 10% will progress within 3 to 6 months of initiating therapy. Such cases are described as showing primary resistance to treatment. Another 40% to 50% of patients will go on to develop resistance within the first two years [25]. Primary resistance is observed in approximately 10% of patients. Tumors that are most likely to show primary resistance are those that are KIT and PDGFRA wild-type, those that have a KIT exon 9 mutation, and those that have a PDGFRA D842V substitution [25]. Delayed imatinib resistance most often is associated with the expansion of tumor clones with secondary KIT or PDGFRA mutations [25]. Our patient was started on imatinib shortly after the postoperative period and has not shown any recurrence or resistance to date, approximately 1.5 years postoperatively.

We present this case to demonstrate a successful management of the largest GIST recorded in literature with invasion into the surrounding structures requiring en bloc resection and a multidisciplinary-staged approach to treatment.
